# Malleatin A and B: New Premyrsine-Type Diterpenes from *Euphorbia malleata* with Cytotoxic Effects Against A2780 Wild and A2780 R-CIS Ovarian Cancer Cell Lines in Mono or Combination Treatment with Cisplatin

**DOI:** 10.5812/ijpr-147396

**Published:** 2024-11-18

**Authors:** Behzad Zolfaghari, Forough Akbari, Sajad Esmaeili, Mahmoud Aghaei, Fatemeh Mosaffa, Seyedeh Sara Ghorbanhosseini, Mustafa Ghanadian

**Affiliations:** 1 Department of Pharmaceutics, Novel Drug Delivery Systems Research Centre, School of Pharmacy, Isfahan University of Medical Sciences, Isfahan, Iran; 2 Isfahan Pharmaceutical Sciences Research center, Isfahan University of Medical Sciences, Isfahan, Iran; 3 Department of Clinical Biochemistry, School of Pharmacy and Pharmaceutical Sciences, Isfahan University of Medical Sciences, Isfahan, Iran; 4 Biotechnology Research Center, Pharmaceutical Technology Institute, Mashhad University of Medical Sciences, Mashhad, Iran; 5 Phytochemistry Research Center, Shahid Beheshti University of Medical Sciences, Tehran, Iran; 6 Department of Phytochemistry, Isfahan Pharmaceutical Sciences Research Center, School of Pharmacy, Isfahan University of Medical Sciences, Isfahan, Iran

**Keywords:** *Euphorbia malleata*, Diterpene, Premyrsinane, Ovarian Cancer, Cisplatin Resistance, Combination Therapy

## Abstract

**Background:**

This study focused on macrocyclic diterpenes derived from Euphorbia, particularly myrsinanes, and their potential in cytotoxic and combination treatments for resistant cancer cells. We examine premyrsinanes isolated from *Euphorbia malleata* and explore their cytotoxic properties.

**Methods:**

*Euphorbia malleata* was collected from Taragh-Roud, Natanz, Iran. The semi-polar chloroform/acetone extract was chromatographed and fractionated using a large silica column. Fractions containing diterpene resonances were selected based on ^1^H-NMR spectra and were further subjected to smaller silica or Sephadex columns, followed by a recycling HPLC system. The isolated compounds were identified through 1D and 2D-NMR experiments and mass spectrometry. The cytotoxicity of the isolated compounds was assessed using the MTT assay against A2780 wild and A2780 cisplatin-resistant (R-CIS) cells, both in mono and combination treatments with cisplatin.

**Results and Conclusions:**

Using a Waters 616 HPLC pump and a YMC prep silica column, we successfully isolated two new premyrsinane diterpenes (Malleatin A and Malleatin B) alongside two known compounds (beta-sitosterol and loliolide). Malleatin A exhibited cytotoxicity against A2780 wild and A2780 R-CIS cells, with an IC_50_ range of 50 - 65 μM in the MTT assay. While cisplatin demonstrated significant cytotoxic effects on the A2780 wild cell line, it was ineffective against the A2780 R-CIS cells due to their resistance. However, the combination therapy of Malleatin A and cisplatin exhibited a synergistic effect, significantly increasing the mortality rate of the resistant cells compared to monotherapy. The Combination Index (CI) of 0.58 indicates effective synergy, and the Dose Reduction Index (DRI) of 3.65 suggests a favorable reduction in the dosage of cisplatin needed, potentially reducing its associated side effects.

## 1. Background

Ovarian cancer is a type of solid tumor that primarily affects women, particularly those who are postmenopausal. It ranks as the sixth most common cancer among women and is the fifth leading cause of cancer-related deaths (1). Initial treatments are ineffective for 20 - 30% of patients due to resistance to single-drug therapy (2). Therefore, it is recommended to use combinations of commonly prescribed drugs, such as carboplatin, with other medications like liposomal doxorubicin, gemcitabine, or taxanes in higher doses to prevent cancer recurrence (3). Furthermore, high doses of these drugs, such as cisplatin used in resistant cases, can lead to various side effects, including nausea and drug intolerance, as well as damage to vital body tissues such as the kidneys, heart, liver, and nervous system.

Researchers have focused on macrocyclic diterpene compounds called myrsinanes derived from *Euphorbia* species, studying their potential anti-cancer properties and effectiveness against multi-drug resistance. These compounds can be categorized into premyrsinanes, myrsinanes, and cyclomyrsinanes. They are biochemically synthesized from jatrophanes and lathyranes (4, 5). These specialized polyoxygenated and polyester compounds are not widely distributed in nature but are commonly found in the *Euphorbiaceae* family, underscoring the biogenetic significance of this plant family in isolating these compounds. Myrsinanes have demonstrated strong cytotoxic effects against phytohemagglutinin-activated T-cell proliferation but only moderate activity against solid cancer cells (4, 5).

In this family, *Euphorbia malleata* is found in Iran's central plateau region within the provinces of Isfahan and Yazd. This plant mainly grows on dry, rocky slopes and foothills at altitudes ranging from 190 to 320 meters (6). In previous studies, researchers identified one triterpene, oleanolic acid; three simple phenolics, methyl gallate, scopoletin, and catechin; and two flavonoids, hyperoside and 5,7,3′,5′-tetrahydroxyflavanone in this plant (7). 

## 2. Objectives

The objective of this study was to isolate polyester macrocyclic diterpenoids and to assess the cytotoxic effects of the isolated diterpenes on both wild-type and resistant A2780 ovarian cancer cells. Additionally, the study evaluated combination indices for their use in combination treatment with cisplatin.

## 3. Methods

### 3.1. Plant Material 

Aerial parts of *Euphorbia malleata* Boiss., also known by the synonym *Tithymalus malleatus* (Boiss.) Soják, were collected in July 2017 during flowering from Taragh-Roud, Natanz County, Isfahan, Iran. The aerial parts were dried in shaded conditions at 25°C. The plant was identified by Joharchi (6), and a voucher specimen numbered SAM-3393 is kept in the Samsam-Shariat Herbarium of the Department of Pharmacognosy at Isfahan University of Medical Sciences. 

### 3.2. Extraction and Isolation 

After drying, the plant material was powdered (5 kg) and extracted by the maceration method using dichloromethane: Acetone (2:1) for two rounds, each lasting four days. The collected extracts were evaporated and concentrated using a rotary evaporator at 45°C. The concentrated extract (220 g, yield = 4.4%) was dissolved in MeOH: H₂O (70:30) and filtered through a reverse silica gel cartridge made from 10% paraffin-impregnated silica gel to remove highly oily and green contents. The resulting clear and brown filtered solution was concentrated to yield 75 g and then fractionated on a silica gel column (40 - 63 µm; 50 × 450 mm) using a gradient solvent system of hexane: Ethyl acetate, which produced 10 subfractions (90:10; 85:15; 80:20; 75:25; 70:30; 65:35; 0:100). 

After checking the TLC profile, which was visualized using 1% cerium sulfate in 5% H₂SO₄, fractions eluted with hexane: Ethyl acetate (85:15, 70:30, and 65:35) that showed pink or light brown spots on the TLC were selected for further analysis. These fractions were submitted to a Sephadex column. Using preliminary ^1^H-NMR spectra, fractions containing terpenoid resonances were selected and injected into an HPLC column (YMC-Silica, 20 × 250 mm) using hexane: Ethyl acetate (65:35) to obtain compound 1 (4 mg) and compound 2 (20 mg) in their pure state. Compounds 3 and 4 were purified by recrystallization from hexane: Acetone. 

### 3.3. Cell Culture 

A2780 cisplatin-resistant (R-CIS) and A2780 Wild ovarian cancer cells, representing R-CIS and non-resistant lines, respectively, were provided by the Mashhad University of Medical Sciences (MUMMS). These cells were cultured in RPMI-1640 media supplemented with FBS, penicillin, and streptomycin (10%, 0.1%, and 0.1%, respectively), and were maintained at 37°C with 5% CO₂ and 95% humidity.

### 3.4. Cell Viability Assay 

The MTT assay for cisplatin and compound 1 was evaluated using the A2780 R-CIS and A2780 wild cells on 96-well plates. A total of 3 × 10³ cells were seeded for 24 hours at 37°C with 5% CO₂. After adding various concentrations of cisplatin (0.1, 1, 5, 10, 50, 100, and 200 µM) or compound 1 (0.1, 1, 10, 20, 40, 100, and 200 µM), the cells were incubated for an additional 48 hours at 37°C in a CO₂ atmosphere. After incubation, 20 µL of MTT reagent (0.5 mg/mL) was added to each well for 4 hours at 37°C, leading to formazan crystal formation. The supernatants were discarded, and the formazan crystals were dissolved in DMSO. The absorbance was read at 570 nm using a Bio-Tek microplate reader (Winooski, VT, USA). Each experiment was conducted in triplicate.

To conduct the combination study in accordance with the study's objectives and the Chou formula (8), it is crucial to maintain a consistent concentration of the tested compound while steadily increasing the concentration of cisplatin. Therefore, we also examined the combination of compound 1 at IC_50_ with cisplatin at varying concentrations of 0.1, 1, 5, 10, 50, 100, and 200 µM for cell viability assays in both cell lines, as previously mentioned.

### 3.5. Combination Effect Analysis 

For the combination treatment, A2780 R-CIS cell lines were treated in a non-constant ratio with a fixed dose of Malleatin A (IC_50_) and varying concentrations of cisplatin, or with a fixed dose of cisplatin (IC_50_) and varying concentrations of Malleatin A for 48 hours. The Combination Index (CI) and Dose Reduction Index (DRI) parameters were calculated according to the Chou equations at the IC_50_ of the combination therapy (8). Combination Index was calculated using the following equation, with the criteria defined as follows: A CI of less than 1 indicates a synergistic effect, a CI equal to 1 indicates an additive effect, and a CI greater than 1 indicates an antagonistic effect.


$$CI=\frac{{IC}_{50}\;of\;cisplatin\;in\;combination\;}{{IC}_{50}\;of\;cisplatin\;alone}+\frac{{IC}_{50}\;of\;malleatin\;in\;combination}{{IC}_{50}\;of\;malleatin\;alone}$$

The DRI was determined using the following equation, which measures how many times the dose of cisplatin could be reduced in combination with Malleatin A compared to its monotherapy. A DRI greater than 1 is considered highly favorable, especially for avoiding high concentrations of chemical drugs and reducing the risk of adverse effects in conditions such as drug resistance.


$$DRI=\frac{{IC}_{50}\;of\;drug\;alone}{{IC}_{50}\;of\;drug\;in\;combination}$$

### 3.6. Statistical Analyses 

Cytotoxicity values were expressed as the mean ± standard deviation. Statistical analyses were performed using one-way analysis of variance, followed by Dunnett's post hoc test, utilizing GraphPad Prism software.

## 4. Results 

**Figure 1.**
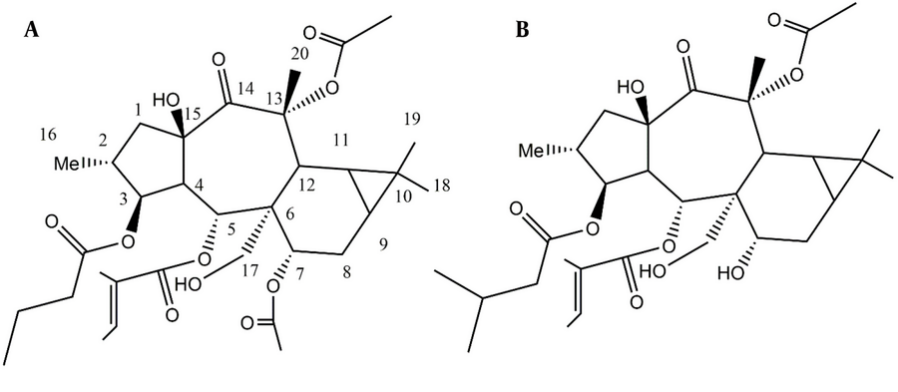
Malleatin A and B from *Euphorbia malleata.*

*Euphorbia malleata* was extracted, and its components were isolated through column chromatography and HPLC, leading to the detection of one steroid, two diterpenes, and one iridoid compound (Figure 1). 

### 4.1. Spectral Data of Isolated Compounds

Compound 1: Colorless oil; for ^1^H and ^13^C NMR data, see Table 1; ESI MASS m/z 621.3 (M+H)^+^.

**Table 1.** ^1^H- and ^13^C NMR Data of Malleatin A (1, CDCl3, 400 MHz for δ_H_; 100 MHz for δ_C_)

Atom	δ_H_ (*J* in Hz)	δ_C_	Atom	δ_H_ (*J* in Hz)	δ_C_
**1a**	1.64 m ^a^	42.99	3-OBut-1'	-	173.07
**1b**	3.17 dd, 13.6, 7.8	-	2'	2.26 m ^a^	36.23
**2**	1.86 m ^a^	37.33	3'	1.56, m ^a^	18.07
**3**	5.28, t, J = 3.5	78.36	4'	0.93, t, j = 7.7	13.88
**4**	2.34, dd, 11.6, 3.5	50.59	5-Tig-1'	-	166.25
**5**	6.27,d, j = 11.3	69.27	2'	-	128.19
**6**	-	48.09	3'	6.53 d, j = 7.2	138.15
**7**	4.9 m ^a^	70.6	4'	1.29, d, j = 7.1	14.08
**8**	1.58 m ^a^, 0.98 m ^a^	22.36	5'	1.47, s	11.97
**9**	0.8 m ^a^	23.98	7-OAc-1'	-	170.17
**10**	-	18.49	2'	2.16, s	21.42
**11**	0.79 m ^a^	19.08	13-OAc-1'	-	170.74
**12**	3.55 m ^a^	35.26	2'	2.13, s	21.4
**13**	-	85.74			
**14**	-	204.39			
**15**	-	84.2			
**16**	0.89, d, j = 6.6	14.03			
**17a**	4.56, d, j = 11.7	64.01			
**17b**	4.94, d, j = 11.7	-			
**18**	1.75	25.01			
**19**	0.97, s	14.97			
**20**	1.08, s	29.54			

^a^ Overlapping with other signals.

Compound 2: Colorless oil; ^1^H-NMR data (400 MHz, CDCl3, J in Hz): δ_H_ 1.57 (m, H-1a), 3.15 (dd, 13.6, 7.9, H-1b), 1.91 (m, H-2), 5.29 (t, j = 3.44, H-3), 2.42 (dd, 11.04, 3.2, H-4), 6.26 (d, j = 11.3, H-5), 3.90 (m, H-7), 1.34-1.24 (m, H-8), 1.06 (m, H-9), 0.81 (m, H-11), 3.39 (m, H-12), 0.92 (m, H-16), 4.50 (d, j = 11.6, H-17a), 4.92 (d, j = 11.8, H-17b), 1.74 (s, H-18), 1.15 (s, H-19), 1.08 (s, H-20), 2.12 (m, 3-OMB-H-2'a), 2.21 (m, 3-OMB-H-2'b), 1.95 (m, 3-OMB-H-3'), 0.94 (d, j = 7.08, 3-OMB-H-4'), 0.91 (d, j = 7.12, 3-OMB-H-5'), 6.67 (d, j = 7.4, 5-Tig-H-3'), 1.38 (d, j = 6.8, 5-Tig-H-4'), 1.52 (s, 5-Tig-H-5'), 2.13 (s, 13-OAC2'). ^13^C NMR data (100 MHz, CDCl3): δ_C_ 43.39 (C1), 37.83 (C2), 79.05 (C3), 49.8 (C4), 70.93 (C5), 49.08 (C6), 66.43 (C7), 24.56 (C8), 24.28 (C9), 18.92 (C10), 20.11 (C11), 34.54 (C12), 86.49 (C13), 204.69 (C14), 84.22 (C15), 14.63 (C16), 64.6 (C17), 25.67 (C18), 15.56 (C19), 30.07 (C20), 172.94 (3-OMB-C1'), 43.14 (3-OMB-C2'), 25.41 (3-OMB-C3'), 23.07 (3-OMB-C4'), 22.89 (3-OMB-C5'), 166.53 (Tig-C1'), 127.95 (5-Tig-C2'), 140.51 (5-Tig-C3'), 14.63 (5-Tig-C4'), 12.16 (5-Tig-C5'), 171.01 (13-OAC1'), 21.77 (13-OAC2'). ESI MASS m/z 593.33 (M+H)^+^.

Compound 3: White solid; ^1^H-NMR data (400 MHz, CDCl3, J in Hz): δ_H_ 1.51 (dd, j = 14.4, 3.6, H-2a), 1.95 (t, j = 2.68, H-2b), 4.32 (m, H-3), 1.75 (dd, j = 13.44, 4.04, H-4a), 1.99 (t, j = 2.68, H-4b), 5.68 (s, H-7), 1.44 (s, H-9), 1.25 (s, H-10), 1.76 (s, H-11), 1.34 (s, 3-OH). ^13^C NMR data (100 MHz, CDCl3): δ_C_ 30.65 (C1), 45.53 (C2), 66.70 (C3), 47.19 (C4), 87.01 (C5), 172.28 (C6), 112.79 (C7), 182.85 (C8), 26.45 (C9), 26.96 (C10), 30.65 (C11). ESI MASS m/z: 415.3 (M+H)^+^.

Compound 4: White solid; ^1^H-NMR data (400 MHz, CDCl3, J in Hz): δ_H_ 3.54 (m, H-3), 5.38 (d, j = 5.2, H-6), 0.69 (s, H-18), 1.03 (s, H-19), 0.95 (d, j = 6.4, H-21), 0.88 (d, j = 7.6, H-24), 0.84 (d, j = 6.8, H-26), 0.84 (d, j = 6.8, H-26), 0.86 (d, j = 6.8, H-27), 0.85 (t, j = 7.4, H-29). ^13^C NMR data (100 MHz, CDCl3): δ_C_ 37.31 (C1), 31.7 (C2), 71.80 (C3), 42.3 (C4), 140.82 (C5), 121.73 (C6), 31.91 (C7), 31.91 (C8), 50.20 (C9), 36.52 (C10), 21.11 (C11), 39.82 (C12), 42.33 (C13), 56.14 (C14), 24.30 (C15), 28.21 (C16), 56.80 (C17), 12.22 (C18), 19.41 (C19), 36.10 (C20), 18.81 (C21), 34.01 (C22), 26.11 (C23), 45.92 (C24), 29.21 (C25), 19.80 (C26), 19.12 (C27), 23.14 (C28), 12.11 (C29). ESI MASS m/z: 197.3 [M + H]^+^.

### 4.2. Cell Viability Assay of Malleatin A and Cisplatin

**Figure 2.**
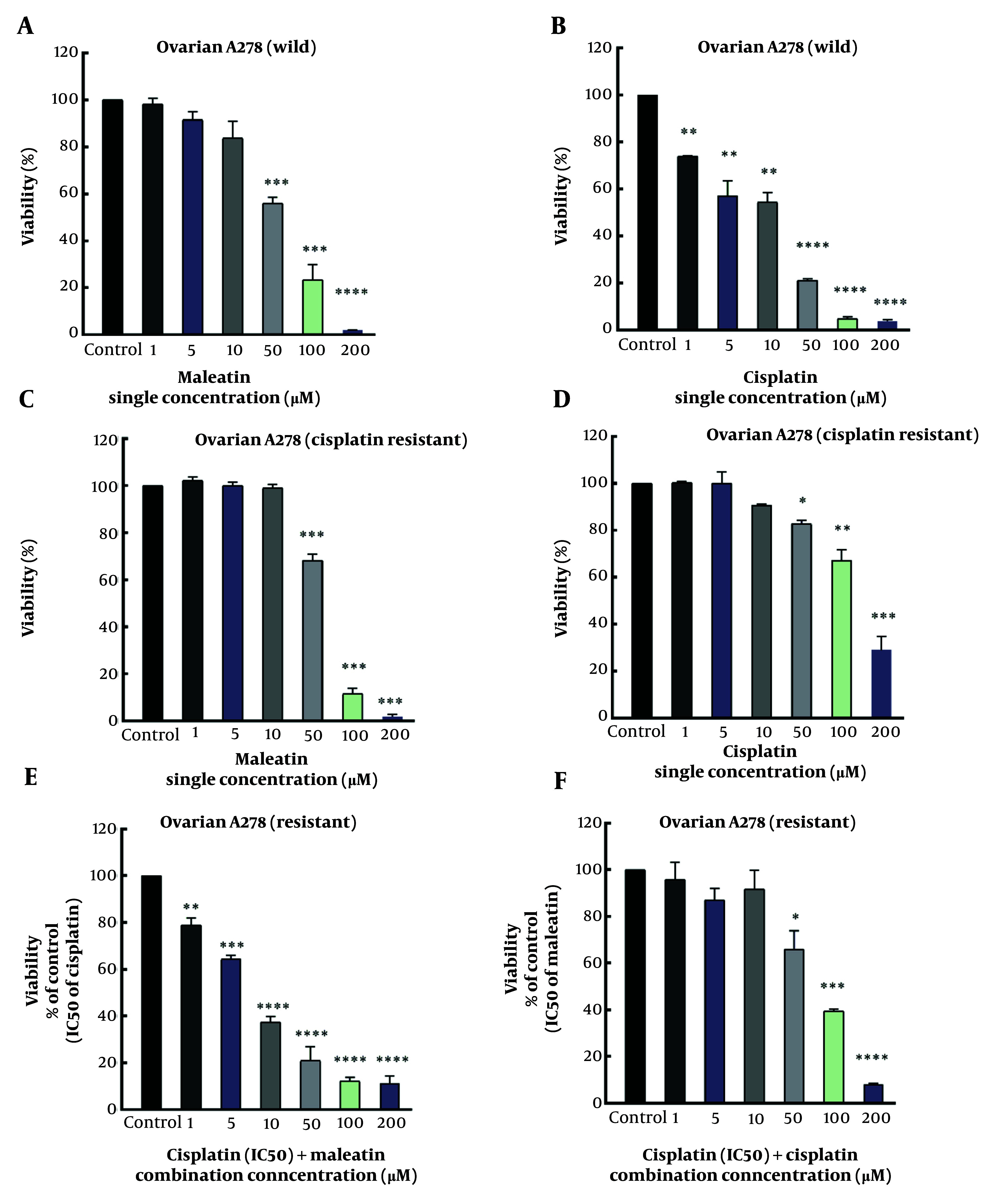
Single treatments of Cisplatin (cis) and Malleatin A (mal) against both A2780 wild-type and A2780 cisplatin-resistant (R-CIS) cells, in addition to combination treatments in resistant ovarian cancer cells. (A, B/C, D), MTT assays for A2780 wild-type/A2780 R-CIS single treatments; (E), Combination treatment of a fixed concentration of cisplatin (IC_50_) while incrementally increasing the concentration of Malleatin A in R-CIS cells; (F), Combination treatment of a fixed concentration of Malleatin A (IC_50_) while incrementally increasing the concentration of cisplatin in R-CIS cells. *P < 0.05, **P < 0.01, ***P < 0.001, ****P < 0.001.

Malleatin A was evaluated against R-CIS and non-resistant ovarian cancer cells to assess its cytotoxicity (Figure 2A and B). Malleatin A demonstrated a cytotoxic effect on the survival of the A2780 wild-type and resistant cell lines, with IC_50_ values of 56.1 ± 2.74 and 62.5 ± 0.7 μM, respectively. Cisplatin exhibited a cytotoxic effect against the survival of the A2780 wild-type and resistant cell lines, with IC_50_ values of 12.1 ± 2.2 and 142.5 ± 10.6 μM (Table 2). 

**Table 2.** IC_50_ Values of Malleatin A and Cisplatin as Single Drugs in A2780 Wild-Type (Non-resistant) and A2780 Cisplatin-Resistant Cell Lines or in Combination in A2780 R-CIS Cells.

Variables and Drugs	Treatment	A2780 Wild Concentration (μM)	A2780 R-CIS Concentration (μM)
**Single**			
Malleatin A	Alone	56.1 ± 2.7	62.5 ± 0.7
Cisplatin	Alone	12.1 ± 2.2	142.5 ± 10.6
**Combination**			
Malleatin A	+ IC_50_ of Cisplatin		18.07 ± 1.5
Cisplatin	+ IC_50_ of Malleatin A		39.45 ± 2.9
**CI**			0.58
**DRI for Cisplatin**			3.65

Abbreviations: CI, Combination Index; DRI, Drug Reduction Index.

### 4.3. Combination Treatment of Malleatin A and Cisplatin

After a 48-hour treatment of A2780 R-CIS cell lines with cisplatin combined with Malleatin A, co-treatment resulted in a significant decrease in cell viability compared to the individual treatments (Figure 2). Figure 2 illustrates that the combination of cisplatin and Malleatin A reduced A2780 R-CIS cell viability more than the single treatments. IC_50_ values are listed in Table 2. 

## 5. Discussion

Using the standard HPLC method on the YMC-silica column, we separated closely related metabolites (1 and 2). Their NMR resonances were characteristic of polyester and polyol diterpene moieties found in *Euphorbia* plants. 

In compound 1, the 13C and 1H spectra revealed a tigliate with chemical shifts at 166.25, 128.19, and 138.15 (δ_H_: 6.53, q, J = 7.2 Hz), 14.08 (δ_H_: 1.29, d, J = 7.1), and 11.97 (δ_H_: 1.47, s), along with a butanoate at 173.07 and 36.23 (δ_H_: 2.26, m), 18.07 (δ_H_: 1.56, m), and 13.88 (δ_H_: 0.93, t, J = 7.7 Hz), as well as two acetate ester groups at 170.17, 21.42 (δ_H_: 2.16, s) and 170.74, 21.40 (δ_H_: 2.13, s) (9). The main structure, devoid of ester groups, contains 20 carbons, including one doublet methyl group at δ_C_: 14.03 (δ_H_: 0.89, d, J = 6.6, Me-16), three singlet methyl groups at δ_C_: 25.01, 14.97, and 29.54 (δ_H_: 1.75, s, Me-18; 0.97, s, Me-19; 1.08, s, Me-20), and three methylene groups at δ_C_: 42.99 (δ_H_: 1.64 m; 3.17 dd, j = 13.6, 7.8, H-1), 22.36 (δ_H_: 1.58 m, 0.98 m, H-8). Additionally, there is one oxygenated carbon at δ_C_: 64.01 (δ_H_: 4.56, d, j = 11.7; 4.94, d, j = 11.7, H-17), eight methines at δ_C_: 37.33 (δ_H_: 1.86 m, H-2), 50.59 (δ_H_: 2.34, dd, j = 11.6, 3.4, H-4), 23.98 (δ_H_: 0.8 m, H-9), 19.08 (δ_H_: 0.79 m, H-11), and 35.26 (δ_H_: 3.55 m, H-12). There are also three oxygenated carbons at δC: 78.36 (δ_H_: 5.28, t, J = 3.5, H-3), 69.27 (δ_H_: 6.27, d, j = 11.3, H-5), and 70.6 (δ_H_: 4.9, overlap, H-7), as well as five quaternary carbons at δ_C_: 48.09 (C6), 18.49 (C10), and two oxycarbons at δ_C_: 78.36 (C13) and 69.27 (C15), along with one ketone group at δ_C_: 204.39 ppm (C14). The DQF-COSY and HSQC-TOCSY spectra assisted in correlating H-1 to H-5 as spin system A and H-7 to H-11 as spin system B in the main structure. Some resonances in the DQF-COSY spectrum overlap; therefore, HSQC-TOCSY was employed to resolve the issue. 

**Figure 3.**
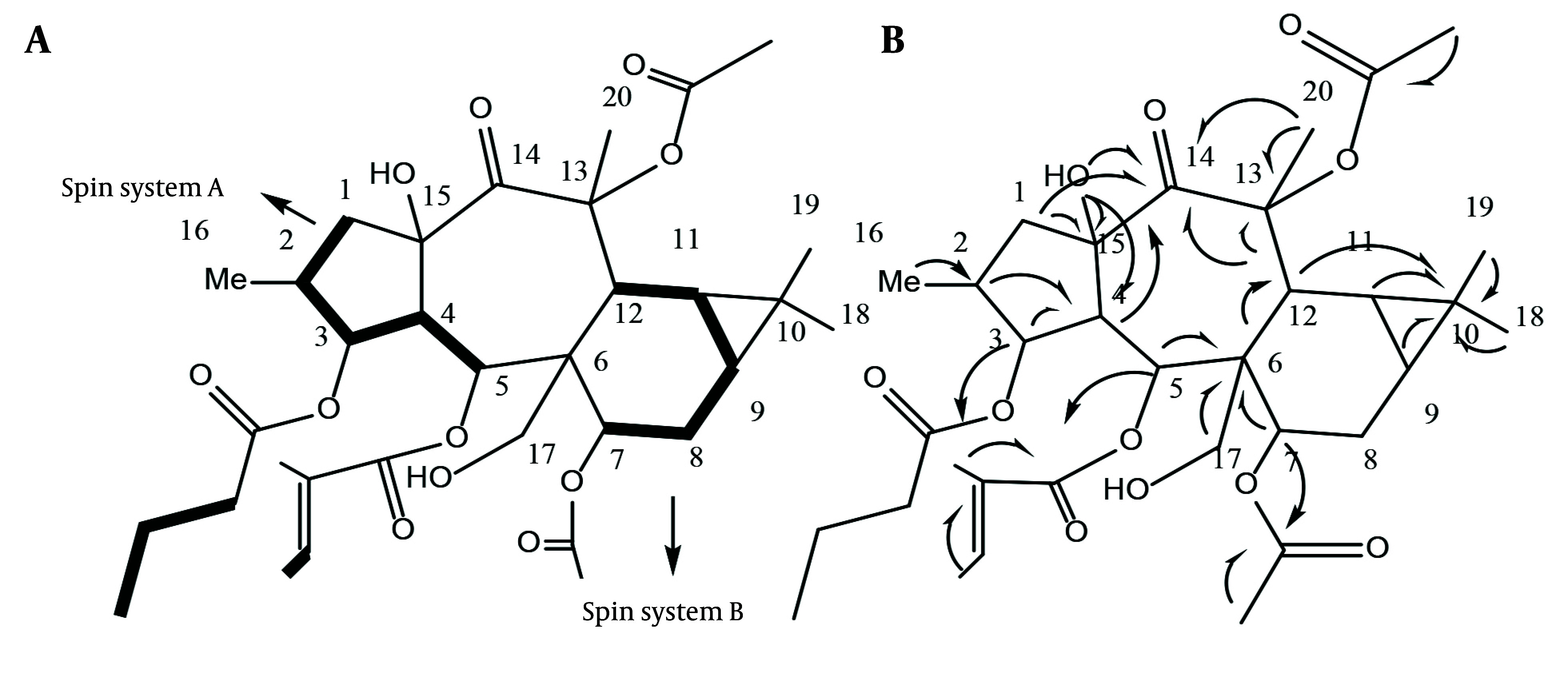
DQFCOSY connections (A) and essential correlations (B) observed in the HMBC spectra of Malleatin A (compound 1).

Heteronuclear Multiple Bond Correlation (HMBC) was utilized to identify correlations up to three bonds. The HMBC of C6 at δ_C_: 48.09 ppm with H5, H7, and H12 correlated spins A and B through C6. The HMBC of C13 at δ_C_: 85.17 ppm with H-12 and Me-20; C14 at δ_C_: 204.39 ppm with H-12, H-1, and H-4; and C10 at δ_C_: 18.49 ppm with Me-18, 19, and C6 with H-17a,b determined the positions of the quaternary carbons C10, C13, C14, C15, as well as the oxymethylene C17, as shown in Figure 3. The HMBC of δ_C_ 173.07 ppm with δ_H_ 5.28 ppm; δ_C_ 166.25 ppm with δ_H_ 6.27 ppm; and δ_C_ 170.17 ppm with δ_H_ 4.9 ppm located the butanoate function on C3, the tigliate ester on C5, and one acetate group on C7. The remaining acetate group did not show HMBC correlations with any of the oxymethins or oxymethylene and is probably attached to one of the quaternary oxycarbons C13 or C15. The HMBC of C15 at δ_C_ 84.2 ppm with the free hydroxy group at δ_H_ 4.38 ppm suggested a hydroxy group on C15, with the remaining acetate ester [δ_C_ 170.74, 21.40 (δ_H_ 2.13, s)] on C13 at δ_C_ 85.74 ppm (Figure 3). 

**Figure 4.**
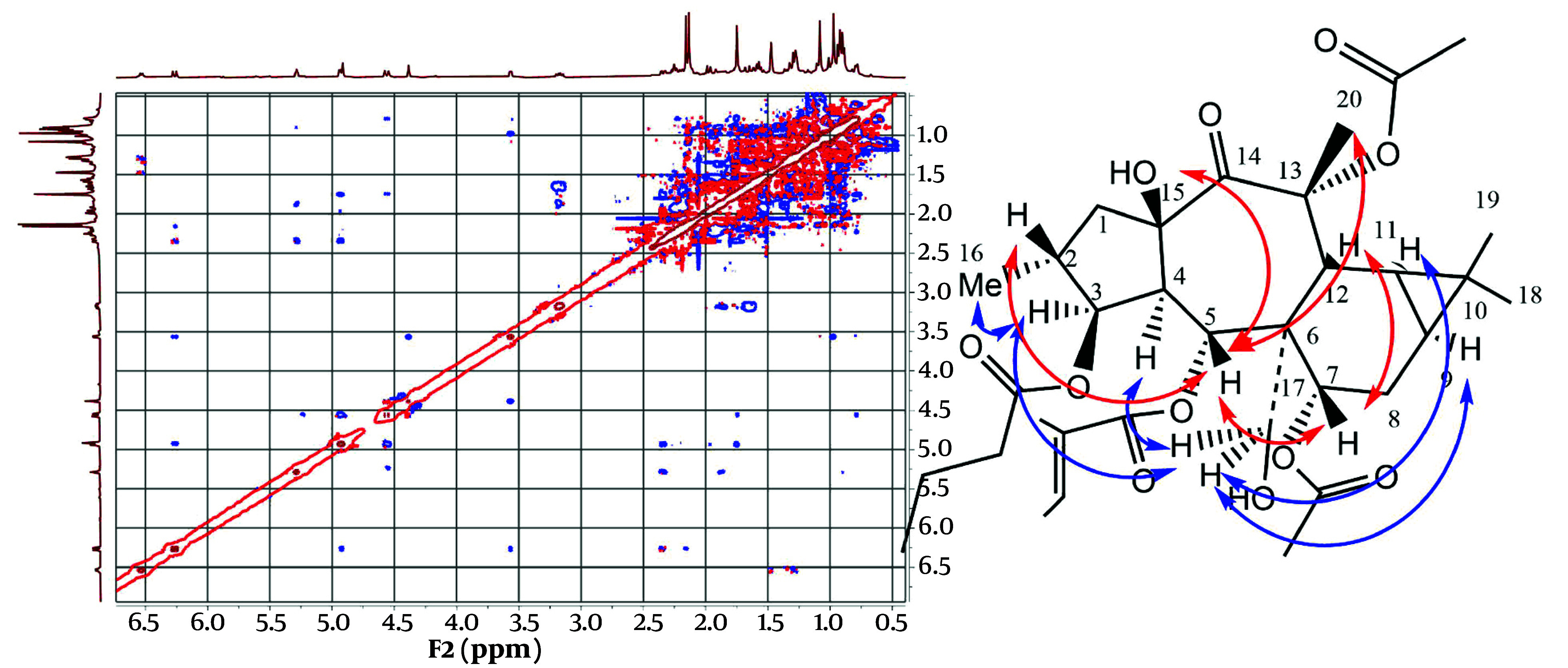
Important correlations observed in the ROESY spectra of Malleatin A (compound 1).

Stereochemistry was determined through a ROESY experiment, taking H-4 in the alpha orientation, as indicated by most of the X-ray crystal structures of myrsinane-type diterpenes reported in the literature (10, 11). As shown in Figure 4, the NOE correlations of H-4/H-17 and H-17/H-3 suggested that H-17 and H-3 are in the alpha position. The NOE correlations of H-17/H-9 and H-11 indicated that they have an alpha-cis configuration and, consequently, a triangular ring in the beta orientation. The NOE correlation of H-3/Me-16 determined that Me-16 is in the alpha position, while H-2 is in the beta orientation. The NOE correlations of H-2 (beta)/H-5; H-5/H-7, Me-20; and H-7/H-12 suggested that H-5, H-7, and H-12 are in the beta position. The NOE correlations of H-5 (beta)/15-OH and H-4 (alpha) indicated that the free hydroxy group is in the beta orientation at C15, with the five-membered ring in the trans position fused to the seven-membered rings in the structure. Finally, compound 1 was proposed as 3β-O-butanoyl-7,13α-O-diacetyl-5α-O-tiglioyl-17α,15β-dihydroxy-14-oxopremyrsinane, a newly described compound named Malleatin A (Figure 4). 

Compound 2 was isolated as an oil. Its NMR resonances exhibited similarities to those of compound 1. The NMR data revealed the presence of three esters attached to the main polyol structure. The NMR data at δ_C_ 166.53, 127.95, and 140.51 (δ_H_: 6.67, q, J = 7.4 Hz), 14.63 (δ_H_: 1.38, s), and 12.16 (δ_H_: 1.52, s) correspond to one tigliate group (9). The NMR data at δ_C_ 172.94 and 43.14 (δ_H_: 2.21, m; 2.12, m), 25.41 (δ_H_: 1.95, m), 23.07 (δ_H_: 0.94, d, j = 7.08), and 22.89 (δ_H_: 0.91) are related to a 3-methylbutanoate (12). The NMR data at δ_C_ 171.01 and 21.77 (δ_H_: 2.13, s) corresponds to one acetyl ester (9). Excluding the esters, the ^13^C NMR and DEPT spectra contained 20 resonances, including four methyl groups, three methylene groups (one of which is oxygenated), eight methine groups (three of which are oxygenated), five quaternary carbons, two oxycarbons, and one ketone group. These data resemble the NMR data of the compound 1 backbone but differ by the loss of one 7-O-acetyl group and the presence of a 3-methylbutanoate ester instead of the butanoate group. The loss of the ester group at C7 causes an upfield NMR shift from δ_C_ 70.60 (δ_H_: 4.9, m) to δ_C_ 66.43 (δ_H_: 3.9, m). The HMBC spectra confirmed the location of the ester groups and determined the structure as 3β-O-3’-methylbutanoyl-13α-O-acetyl-5α-O-tiglioyl-17α,15β-dihydroxy-14-oxopremyrsinane, a newly described compound named Malleatin B (Figure 1). Compounds 3 and 4 were identified as beta-sitosterol and Loliolide, with the C11 iridoid structure, as known compounds consistent with literature data (13, 14).

In the MTT assay, A2780 wild and A2780 R-CIS cell lines were treated with Malleatin A and cisplatin in monotherapy. In the A2780 wild cell line, at lower concentrations of 1, 5, and 10 μM, the cytotoxic activity of Malleatin A was insignificant. However, at higher concentrations of 50, 100, and 200 μM, cytotoxicity was significant, with P-values less than 0.001. Cisplatin exhibited significant cytotoxicity at a lower concentration of 1 μM (P < 0.01). In the A2780 R-CIS cell line, at lower concentrations of 1, 5, and 10 μM, the cytotoxic activity of Malleatin A was not significant, but it became significant at higher concentrations (P < 0.001). The A2780 R-CIS cells demonstrated resistance to cisplatin's cytotoxic activity at lower concentrations of 1, 5, and 10 μM, with no significant reduction in survival. In contrast, at higher concentrations of 50, 100, and 200 μM, cytotoxicity was significant, with P-values less than 0.05, 0.01, and 0.001, respectively, which were significantly lower than those observed in the nonresistant wild type.

Combination therapy was conducted only on the resistant cells. A2780 R-CIS cell lines were treated in two ways: With a fixed concentration of Malleatin A (IC_50_ value) combined with varying concentrations of cisplatin for 48 hours, or with a fixed concentration of cisplatin (IC_50_ value) combined with different concentrations of Malleatin A for 48 hours, separately. Co-treatment of A2780 R-CIS cell lines with Malleatin A and cisplatin resulted in a significantly increased mortality rate of the A2780 R-CIS cell lines compared to when cisplatin was used alone. Figure 2 illustrates that the combined treatment of cisplatin and Malleatin A reduced the proliferation of A2780 R-CIS cell lines more effectively than cisplatin alone, lowering their respective IC_50_ values, as shown in Table 2. The CI and DRI values against the A2780 cells were calculated using the Chou equation (8). For example, in combination treatments of cisplatin with a fixed dose of Malleatin A against A2780 R-CIS cells, the CI was 0.58, indicating synergistic effects.

The DRI measures how much the dose of cisplatin in a combination can be reduced, which is highly important due to its severe side effects. A DRI of 1 indicates no reduction in the drug dose within the combination. A DRI greater than 1 indicates a favorable dose reduction, while a DRI less than 1 suggests an unfavorable dose increase. The DRI value of cisplatin in combination with a fixed dose of Malleatin A against A2780 R- CIS was 3.65, which was favorable (Table 2). It indicates that 3.65 fold the dose of cisplatin is reduced compared to monotreatment in resistant cells.

The cytotoxic effects of Malleatin A were consistent with previous findings regarding the cytotoxic properties of other myrsinane compounds. In a study by Abdolmohammadi et al. (as cited by Mendes et al.), 13(17)-Epoxy-8,10(18)-Myrsinadiene polyester compounds demonstrated cytotoxicity against OVCAR-3 (HTB-161) and Caov-4 (CVCL_0202) ovarian cancer cells, with IC_50_ values ranging from 35 to 40 µM (15). Proliferin A, featuring a 13(17)-Epoxy-8-myrsinene structure isolated from *E. prolifera*, exhibited cytotoxicity against A2780 wild cells with an IC_50_ of 7.7 µM (16). Previous research has reported MDR activity in *Myrsinane*-type compounds, with some myrsinanes displaying MDR activity by inhibiting the rhodamine 123 efflux pump in mouse lymphoma cells (15). Additionally, a study by Vasas et al. explored the synergistic activity of myrsinanes in combination therapy with doxorubicin (17). 

### 5.1. Limitations of the Study 

Some fractions with low diterpenoid contents were not determined.

### 5.2. Conclusions 

The aerial flowering parts of *E. malleata* were analyzed for phytochemicals, resulting in the isolation of two new premyrsinane diterpenes, Malleatin A and B, both featuring a 4-oxopremyrsinane 3β,5α,7,13,17α,15β-hexahydroxy structure. Malleatin A demonstrated cytotoxicity against both A2780 wild and A2780 R-CIS cells, with an IC50 in the range of 50 - 65 μM in the MTT assay. Cisplatin exhibits significant cytotoxicity against the A2780 wild cell line but alone was not sufficient to overcome the resistance exhibited by the A2780 R-CIS cells. However, combination therapy utilizing Malleatin A and cisplatin yields promising results, as evidenced by a synergistic effect that enhances the mortality rate of resistant A2780 R-CIS cells compared to monotherapy. The calculated CI of 0.58 suggests effective synergy, while the DRI of 3.65 indicates a favorable reduction in the required dose of cisplatin, potentially mitigating its side effects. These findings highlight the therapeutic potential of Malleatin A in enhancing the efficacy of cisplatin treatment in resistant ovarian cancer cells, warranting further investigation into combination regimens for improved clinical outcomes.

## Data Availability

The dataset presented in this study is available upon request from the corresponding author during submission or after publication.
